# Supplementary material for: Structural assessment of HLA-A2-restricted SARS-CoV-2 spike epitopes recognized by public and private T-cell receptors
Source: Nat Commun. 2022 Jan 10;13:19. doi: 10.1038/s41467-021-27669-8 (PMC8748687; doi:10.1038/s41467-021-27669-8)
Supplement: Supplementary file 1 — Supplementary Information [file 41467_2021_27669_MOESM1_ESM.pdf]

Supplementary Information

Structural assessment of HLA-A2-restricted SARS-CoV-2 spike epitopes recognised by public and private T-cell receptors

Daichao Wu et al.

Supplementary Tables 1–11

Supplementary Figures 1–7

Supplementary References

Supplementary Table 1. SARS-CoV2-specific TCR germline genes and CDR3 sequences

Receptor	α chain				β chain			
	TRAV	CDR3α	TRAJ	Patients from (26)	TRBV	CDR3β	TRBJ	Patients from (26)
RLQ1	TRAV25	CAGDSNDYKLSF	TRAJ20	p1434	TRBV11-2	CASSLGGAGGADTQYF	TRBJ2-3	p1434
RLQ2	TRAV25	CAGDSNDYKLSF	TRAJ20	p1434	TRBV6-5	CASSYEKGIMNTEAFF	TRBJ1-1	p1434
**RLQ3**	**TRAV16**	**CALSGFNNAGNMLTF**	**TRAJ39**	**p1434**	**TRBV11-2**	**CASSLGGAGGADTQYF**	**TRBJ2-3**	**p1434**
RLQ4	TRAV16	CALSGFNNAGNMLTF	TRAJ39	p1434	TRBV6-5	CASSYEKGIMNTEAFF	TRBJ1-1	p1434
**RLQ5**	**TRAV12-2**	**CALSSNDYKLSF**	**TRAJ20**	**p1437 p1499**	**TRBV6-5**	**CATTERDQETQYF**	**TRBJ2-5**	**p1437**
**RLQ7**	**TRAV38-2DV8**	**CASSGNTPLVF**	**TRAJ29**	**p1445**	**TRBV12-3**	**CASTWGRASTDTQYF**	**TRBJ2-3**	**p1445**
**RLQ8**	**TRDV1**	**CALGERDSWGKFQF**	**TRAJ24**	**p1448**	**TRBV20-1**	**CSALTPALAGGVPETQYF**	**TRBJ2-5**	**p1448**
**YLQ7**	**TRAV12-2**	**CAVNRDDKIIF**	**TRAJ30**	**p1434 p1437 p1445 p1449 p1480 p1484 p1495**	**TRBV7-9**	**CASSPDIEQYF**	**TRBJ2-7**	**p1434 p1437 p1445 p1480 p1484**

TCR sequences with confirmed epitope specificity are shown in **bold**.

Supplementary Table 2. Affinity of TCRs for SARS-CoV-2 spike epitopes and epitope variants

Virus	Epitope	Identity with RLQ/YLQ	Sequence	YLQ7	RLQ3	RLQ5	RLQ7	RLQ8
SARS-CoV-2	RLQ	9/9	RLQSLQTYV		32.9 µM	3.4 µM	49.0 µM	9.7 µM
MERS	RLT	4/9	RLTTLNAFV		NB			
SARS	RLQ	9/9	RLQSLQTYV		32.9 µM			
HKU1	RLT	5/9	RLTALNAYV		NB			
OC43	RLT	5/9	RLTALNAYV		NB			
NL63	RLA	4/9	RLAALNAFV		NB			
229E	RLA	4/9	RLAALNVFV		NB			
SARS-CoV-2 variant	T1006I	8/9	RLQSLQ**I**YV		170 µM	23.0 µM	62.8 µM	>50 µM
SARS-CoV-2	YLQ	9/9	YLQPRTFLL	1.8 µM				
MERS	KLQ	7/9	KLQPLTFLL	NB				
SARS	YLK	6/9	YLKPTTFML	NB				
HKU1	PLS	4/9	PLSKRQYLL	NB				
OC43	PLT	4/9	PLTSRQYLL	NB				
SARS-CoV-2 variant	P272L	8/9	YLQ**L**RTFLL	116 µM				

Affinities were measured by SPR. NB, no binding.

Supplementary Table 3. YLQ and RLQ epitope variants in SARS-CoV-2 sequences

Substitution	Sequence	Frequency[a]
P272L	YLQ**L**RTFLL	0.59%
T1006I	RLQSLQ**I**YV	0.04%
R273S	YLQP**S**TFLL	0.011%
T274I	YLQPR**I**FLL	0.007%
R273K	YLQP**K**TFLL	0.006%
P272H	YLQ**H**RTFLL	0.006%
P272S	YLQ**S**RTFLL	0.005%
T274N	YLQPR**N**FLL	0.003%
Q271K	YL**K**PRTFLL	0.003%
Q1005H	RLQSL**H**TYV	0.003%
L270F	Y**F**QPRTFLL	0.0002%

[a]Frequency of substitution in SARS-CoV-2 spike glycoprotein sequences in the GISAID database (www.gisaid.org).

Supplementary Table 4. Data collection and refinement statistics

	RLQ3	RLQ–HLA-A2	RLQ3–RLQ–HLA-A2	YLQ7	YLQ–HLA-A2	YLQ7–YLQ–HLA-A2
PDB	7N1C	7N1B	7N1E	7N1D	7N1A	7N1F
**Data collection**						
Resolution range (Å)	46.6–1.88 (1.95–1.88)	43.2–2.81 (2.91–2.81)	49.4–2.30 (2.38–2.30)	38.8–2.35 (2.43–2.35)	43.3–2.06 (2.14–2.06)	48.6–2.39 (2.48–2.39)
Space group	*P*212121	*P*1	*P*1211	*I*4	*P*3212	*C*121
Unit cell parameters	42.1, 101.9, 104.7 90, 90, 90	47.2, 49.3, 117.1 91.9, 92.4, 118.6	52.8, 68.2, 148.6 90, 94.6, 90	109.8, 109.8, 99.6 90, 90, 90	85.1, 85.1, 214.2 90, 90, 120	225.3, 49.1, 91.5 90, 91.8, 90
Total reflections[a]	239,229 (21,569)	37,697 (3,276)	159,246 (15,350)	172,549 (17,833)	558,646 (56,058)	133,860 (12,215)
Unique reflections[a]	37,372 (3,632)	21,652 (2,067)	46,769 (4,435)	24,641 (2,476)	54,889 (5,464)	39,704 (3,697)
Multiplicity[a]	6.4 (5.9)	1.7 (1.6)	3.4 (3.5)	7.0 (7.2)	10.2 (10.3)	3.4 (3.3)
Completeness (%)[a]	99.8 (98.6)	96.1 (89.1)	99.2 (94.8)	99.9 (100.0)	100 (99.9)	99.0 (92.4)
Mean $I/\sigma(I)$[a]	18.6 (2.9)	9.4 (5.8)	12.6 (3.0)	12.8 (2.9)	17.8 (5.6)	9.4 (2.1)
Wilson *B* factor (Å^2)	32.6	25.6	32.2	44.9	28.6	48.3
R_{merge}[a,b]	0.067 (0.399)	0.128 (0.320)	0.148 (0.472)	0.108 (0.549)	0.141 (0.427)	0.065 (0.363)
CC1/2	0.996 (0.937)	0.911 (0.638)	0.952 (0.753)	0.994 (0.832)	0.991 (0.958)	0.993 (0.938)
**Refinement**						
Reflections used in refinement[a]	37,368 (3,631)	21,643 (2,066)	46,625 (4,411)	24,640 (2,476)	54,879 (5,464)	39,630 (3,680)
R_{work}[c]	0.201 (0.256)	0.211 (0.256)	0.213 (0.277)	0.204 (0.265)	0.196 (0.226)	0.199 (0.285)
R_{free}[c]	0.231 (0.280)	0.269 (0.339)	0.259 (0.369)	0.271 (0.377)	0.236 (0.280)	0.235 (0.362)
No. of protein atoms	3,462	6,261	6,631	3,473	6,289	6,567
No. of waters	267		253	142	482	87
Protein residues	440	764	826	439	766	818
r.m.s.d. from ideality						
Bond lengths (Å)	0.009	0.005	0.010	0.008	0.009	0.009
Bond angles (º)	1.44	1.09	1.30	1.30	1.25	1.26
Ramachandran plot statistics						
Favored (%)	96.1	96.1	95.1	95.9	97.6	96.2
Allowed (%)	3.7	3.5	4.7	3.9	2.4	3.5
Disallowed (%)	0.2	0.4	0.3	0.2	0.0	0.4
Rotamer outliers (%)	1.9	0	0.8	0.3	0.9	1.6
Clashscore	6.0	8.1	9.3	8.9	7.7	8.0
Average *B* factor (Å^2)	41.4	31.2	41.1	47.5	35.4	62.6
Protein	41.3	31.2	41.2	47.5	35.2	62.7
Waters	42.2		40.5	47.6	38.0	53.4

[a]Values in parentheses correspond to the highest resolution shell.

[b]$R_{merge} = \sum|I_j - \langle I \rangle|/\sum I_j$, where I_j is the intensity of an individual reflection and $\langle I \rangle$ is the average intensity of that reflection.

[c]R_{work} (R_{free}) = $\sum||F_o| - |F_c||/\sum|F_o|$; 5.0% of data were used for R_{free}.

Supplementary Table 5. Interactions between RLQ3 and HLA-A2

HLA-A2	RLQ3		
	Hydrogen bonds	Van der Waals contacts	Water bridges
α1			
K66H		N92α(2)	
V76H		N49β(3),	
T80H	N49β(Nδ2) T80H(Oγ1),	N49β(2),	
α2			
H145H		L94β(2)	
K146H		G26β(1),<br>L94β(3),<br>G96β(2)	G95β(N) S253 K146H(O),<br>G96β(N) S253 K146H(O),<br>D101β(Oδ2) S253 K146H(O)
W147H	G96β(O) W147H(Nε1),	G96β(3)	
A149H		L94β(1),<br>D101β(2)	
A150H	R48α(Nη2) A150H(O),	R48α(3),<br>A100β(1),<br>D101β(5)	A100β(O) S71 A150H(O),
H151H	S51α(Oγ) H151H(Nε2),	I50α(1),<br>S51α(4)	
E154H		I50α(1)	
Q155H	E31α(Oε2) Q155H(Nε2),<br>R48α(Nη1) Q155H(Nε2),	E31α(3),<br>R48α(3),<br>I50α(3),<br>F91α(4)	P30α(O) S68 Q155H(Oε1, Nε2),<br>E31α(Oε1) S68 Q155H(Oε1, Nε2)

Contact residues were identified with the CONTACT program (1). Hydrogen bonds were calculated using a cut-off distance of 3.5 Å. The cut-off distance for van der Waals contacts was 4 Å.

Supplementary Table 6. Interactions between RLQ3 and RLQ peptide

RLQ	RLQ3		
	Hydrogen bonds	Van der Waals contacts	Water bridges
S4p	N92α(Nδ2) S4p(Oγ)	S29α(4), F91α(2) N92α(2)	
L5p		F91α(9), N96α(2) A100β(1)	
Q6p	F91α(O) Q6p(Nε2), N93α(O) Q6p(Nε2), G95α(N) Q6p(Oε1), N96α(Nδ2) Q6p(N), N96α(Oδ1) Q6p(N), N96α(Nδ2) Q6p(O), N96α(Oδ1) Q6p(Nε2),	F91α(2), N93α(3), A94α(2), G95α(3), N96α(9) A97β(3)	
T7p		G96β(4)	
Y8p	G96β(O) Y8p(N), G96β(O) Y8p(O),	Q48β(11), V53β(1), G95β(3), G96β(6), G98β(2),	Y8p(Oη) S106 Q46β(Nε2), Y8p(Oη) S106 Q48β(Oε1)

Contact residues were identified with the CONTACT program (1). Hydrogen bonds were calculated using a cut-off distance of 3.5 Å. The cut-off distance for van der Waals contacts was 4 Å.

Supplementary Table 7. Predicted TCR binding energy changes from computational alanine scanning of peptide positions in SARS-CoV-2 TCR–pMHC complexes.

**RLQ3–RLQ–HLA-A2**		**YLQ7–YLQ–HLA-A2**	
**Mutation**	**ΔΔ*G*[1]**	**Mutation**	**ΔΔ*G*[1]**
R1A	0	Y1A	0
L2A	0	L2A	0
Q3A	0	**Q3A**	**1.3**
S4A	-0.1	P4A	0.1
**L5A**	**1.0**	**R5A**	**3.0**
**Q6A**	**2.3**	**T6A**	**1.2**
T7A	0.2	F7A	0.2
**Y8A**	**1.9**	L8A	0.7
V9A	0	L9A	0

[1]TCR binding energy changes for individual peptide residue alanine mutations, calculated using Rosetta with experimentally determined TCR–pMHC complex structures as input. Values are in Rosetta Energy Units (REU), which correspond to energy in kcal/mol. Substitutions with high predicted ΔΔ*G*s (≥ 1 REU), reflective of likely energetic hotspots, are in bold.

Supplementary Table 8. Predicted HLA-A2 binding affinities and TCR binding effects for epitope variants across coronaviruses and within SARS-CoV-2

Lineage[1]	Accession ID[2]	Virus/ Variant	Identity[3]	Sequence	Pred. HLA-A2 affinity (nM)[4]	Predicted TCR ΔΔG[5]	Measured TCR ΔΔG, kcal/mol[6]
RLQ epitope orthologs							
B, clade 1b	QHD43416.1	SARS-CoV-2	9	RLQSLQTYV	11.92	0	
B, clade 1b	QHR63300.2	RaTG13	9	RLQSLQTYV	11.92	0	
B, clade 1b	QIG55945.1	GD_pangolin	9	RLQSLQTYV	11.92	0	
B, clade 1a	AYV99761.1	SARS-CoV	9	RLQSLQTYV	11.92	0	
B, clade 1a	QJE50589.1	SHC014	9	RLQSLQTYV	11.92	0	
B, clade 1a	AGZ48828.1	WIV1	9	RLQSLQTYV	11.92	0	
B, clade 1a	QND76034.1	HKU3	9	RLQSLQTYV	11.92	0	
B, clade 1a	AHX37569.1	LYRa3	9	RLQSLQTYV	11.92	0	
B, clade 2	EPI_ISL_412977	RmYN02	9	RLQSLQTYV	11.92	0	
B, clade 2	ATO98120.1	rs4081	9	RLQSLQTYV	11.92	0	
B, clade 2	ABD75323.1	Rf1	9	RLQSLQTYV	11.92	0	
B, clade 3	APO40579.1	BtKY72	9	RLQSLQTYV	11.92	0	
B, clade 3	YP_003858584.1	BM48-31	9	RLQSLQTYV	11.92	0	
A	YP_173238.1	HKU1	5	RLTALNAYV	8.27	1.6	NB
A	ANZ78843.1	OC43	5	RLTALNAYV	8.27	1.6	
A	ACN89742.1	MHV-1	5	RLTALNAYV	8.27	1.6	
A	YP_009113025.1	HKU24	5	RLTALNAYV	8.27	1.6	
C	YP_009047204.1	MERS	4	RLTTLNAFV	13.32	1.6	
C	YP_001039953.1	HKU4	5	RLISLNAFV	4.97	2.2	NB
C	YP_001039962.1	HKU5	5	RLTSLNAFV	10.18	2.2	
C	AGC51116.1	KW2E-F93	4	RLTTLNAFV	13.32	1.6	
C	QGA70692.1	HKU31	5	RLTSLNAFV	10.18	2.2	
D	YP_001039971.1	HKU9	5	RMMVLNTYV	3.76	1.5	
D	ADX59466.1	KY24	5	RMVVLNTYV	21.45	1.6	
D	QDF43840.1	GX2018	5	RTVVLNTYV	841.78	1.6	
Hibecovirus	YP_009072440.1	Zhejiang2013	7	RLQVLQTFV	36.54	0.8	NB
alpha	AFV53148.1	NL63	4	RLAALNAFV	5.36	2.2	NB
alpha	ABB90529.1	229E	4	RLAALNVFV	5.01	2.1	
YLQ epitope orthologs							
B, clade 1b	QHD43416.1	SARS-CoV-2	9	YLQPRTFLL	4.3	0	
B, clade 1b	QHR63300.2	RaTG13	9	YLQPRTFLL	4.3	0	
B, clade 1b	QIG55945.1	GD_pangolin	7	YLAPRTFML	3.01	1.9	1.7
B, clade 1a	AYV99761.1	SARS-CoV	6	YLKPTTFML	12.25	4.6	NB
B, clade 1a	QJE50589.1	SHC014	6	YLKPTTFML	12.25	4.6	NB
B, clade 1a	AGZ48828.1	WIV1	6	YLKPTTFML	12.25	4.6	NB

B, clade 1a	QND76034.1	HKU3	4	NLKYSTFML	616.38	3.5	
B, clade 1a	AHX37569.1	LYRa3	6	YLKPTTFML	12.25	4.6	NB
B, clade 2	ATO98120.1	rs4081	4	NLKYSTFML	616.38	3.5	
B, clade 2	ABD75323.1	Rf1	4	NLKQSTFML	756.46	3.2	
B, clade 2	EPI_ISL_412977	RmYN02	3	SLKLTTIML	1121.94	4.2	
B, clade 3	APO40579.1	BtKY72	4	HLKPLTMLA	1682.36	3.5	
B, clade 3	YP_003858584.1	BM48-31	4	HLKPLTMLV	157.7	3.5	
A	YP_173238.1	HKU1	4	PLSKRQYLL	7176.36	1	
A	ANZ78843.1	OC43	4	PLTSRQYLL	5129.13	1.8	
A	ACN89742.1	MHV-1	3	PLVERQYLF	12445.47	0.6	
A	YP_009113025.1	HKU24	4	PLIKREYLL	2115.53	1.6	
C	YP_009047204.1	MERS	7	KLQPLTFLL	6.12	2.3	NB
C	YP_001039953.1	HKU4	4	KLHQLTYLL	14.83	4.1	
C	YP_001039962.1	HKU5	5	KLHPLTYLL	9.27	5.1	
C	AGC51116.1	KW2E-F93	4	YLYELPYLL	1.93	12.8	
C	QGA70692.1	HKU31	2	QLHKLNYLV	59.62	5.2	
D	YP_001039971.1	HKU9	3	HLINRDLLV	49.64	2.3	
D	ADX59466.1	KY24	2	PLSKQDVLV	6784.7	4.2	
D	QDF43840.1	GX2018	2	PIVQRELLV	17412.75	1.5	
Hibecovirus	YP_009072440.1	Zhejiang2013	3	QLKKSTFMF	14082.76	3.1	
alpha	AFV53148.1	NL63	1	AFATFVDVL	10799.6	5.8	NB
alpha	ABB90529.1	229E	2	ALASYADVL	147.99	9	NB
SARS-CoV-2 Variants							
-	-	T1006I	8	RLQSLQIYV	6.57	0.1	0.77
-	-	Q1005H	8	RLQSLHTYV	14.92	1.3	
-	-	L270F	8	YFQPRTFLL	1114.21	0	
-	-	P272L	8	YLQLRTFLL	9.56	-0.2	2.5
-	-	T274N	8	YLQPRNFLL	4.73	1.2	
-	-	T274I	8	YLQPRIFLL	3.21	1.3	
-	-	R273S	8	YLQPSTFLL	3.56	2.6	
-	-	Q271K	8	YLKPRTFLL	16.03	1.0	
-	-	R273K	8	YLQPKTFLL	4	2.9	
-	-	P272H	8	YLQHRTFLL	5.65	0.0	
-	-	P272S	8	YLQSRTFLL	4.65	-0.2	

[1]Betacoronavirus lineage (A-D, or Hibecovirus), or “alpha” in the case of alphacoronaviruses. For lineage B (sarbecoviruses), which includes SARS-CoV-2 and SARS-CoV, clade information is also provided. “-“: SARS-CoV-2 variant.
[2]NCBI Genbank or GISAID accession ID.
[3]Number of residues identical with SARS-CoV-2 YLQ or RLQ epitope.
[4]Predicted HLA-A*02:01 binding (IC_{50}, nM), from NetMHCPan 4.1 (2).
[5]Predicted RLQ3 or YLQ7 TCR binding affinity change ($\Delta\Delta G$) for altered peptide versus RLQ or YLQ epitope, calculated using Rosetta (3) with RLQ3-RLQ-HLA-A2 or YLQ7-YLQ-HLA-A2 complex structures. Units are Rosetta Energy Units, which are comparable to kcal/mol.

[6]Measured RLQ3 or YLQ7 TCR binding affinity change versus RLQ or YLQ epitope, from this study. NB: no measurable binding detected.

Supplementary Table 9. Interactions between YLQ7 and HLA-A2

HLA-A2	YLQ7		
	Hydrogen bonds	Van der Waals contacts	Water bridges
α1			
R65H	R65H(Nε) D94α(Oδ2)	D94α(4)	
K66H		D94α(1)	
A69H			A69H(O) S66 Q50β(Nε2)
Q72H		L55β(3)	
T73H		R31β(1), Q50β(2)	T73H(Oγ1) S66 Q50β(Nε2)
V76H		N51β(1)	
α2			
A150H			A150H(O) S53 Y51α(Oη),
H151H		F49α(4),	
E154H	S52α(Oγ) E154H(Oε2)	Y51α(3), S52α(4)	E154H (Oε2) S41 Y51α(N) E154H (O) S16 S52α(Oγ)
Q155H	Q31α(Oε1) Q155H(O), S32α(Oγ) Q155H(Oε1),	Q31α(1), S32α(2), Y51α(10) I98β(1)	
R157H	S52α(Oγ) R157H(Nη2)	S52α(2)	R157H (NH2) S16 S52α(Oγ)
A158H		Y51α(1), K67α(1)	
Y159H		Q31α(1)	
E166H	R28α(Nε) E166H(Oε1), R28α(Nε) E166H(Oε2),	R28α(5)	

Contact residues were identified with the CONTACT program (1). Hydrogen bonds were calculated using a cut-off distance of 3.5 Å. The cut-off distance for van der Waals contacts was 4 Å.

Supplementary Table 10. Interactions between YLQ7 and YLQ peptide

YLQ	YLQ7		
	Hydrogen bonds	Van der Waals contacts	Water bridges
Y1p		D27α(1), G29α(1)	
Q3p	Q31α(Oε1) Qp3(Nε2)	Q31α(4)	
P4p		G29α(1), Q31α(2) D94α(5)	P4p(O) S35 D95α(Oδ2)
R5p	Q31α(Nε2) R5p(N) S32α(Oγ) R5p(Nε) D95α(Oδ1) R5p(Nη1) D97β(Oδ1) R5p(Nη2) D97β(Oδ2) R5p(Nη2) D97β(Oδ1) R5p(Nη1)	Q31α(2) S32α(1), N92α(2), D94α(2) D95α(5) R31β(1) D97β(6) I98β(8)	
T6p	D95α(Oδ1) T6p(N), D95α(Oδ2) T6p(N), D95α(Oδ1) T6p(O), D95α(Oδ2) T6p(Oγ1) R31β(Nη1) T6p(O), R31β(Nη1) T6p(Oγ1), R31β(Nη2) T6p(O), D97β(Oδ1) T6p(O)	D95α(9) R31β(2)	
F7p		D97β(4)	
L8p		N30β(1) R31β(2) Q50β(3)	

Contact residues were identified with the CONTACT program (1). Hydrogen bonds were calculated using a cut-off distance of 3.5 Å. The cut-off distance for van der Waals contacts was 4 Å.

Supplementary Table 11. Sequences of TCR genes codon-optimized for expression in *E. coli*

TCR gene name	Nucleotide sequence
RLQ3<br>alpha	CAT**ATG**CAGCGTGTGACCCAGCCGGAAAAACTGCTGAGCGTGTTTAAAGGCGCGCCGGTTGAACT<br>GAAATGCAATTATAGCTATAGCGGCAGCCCGGAACTGTTTTGGTATGTGCAGTATAGCCGCCAGC<br>GCCTGCAGCTGTTGCTGCGCCATATTAGCCGTGAAAGCATTAAAGGCTTTACCGCGGATCTGAAC<br>AAAGGCGAAACCAGCTTTCATCTGAAAAAACCGTTTGCGCAGGAAGAGGATAGCGCGATGTATTA<br>CTGCGCGCTGAGCGGCTTTAACAATGCGGGTAACATGCTGACCTTTGGTGGCGGTACCCGTCTGA<br>TGGTGAAACCGAACATTCAGAACCCGGATCCGGCGGTTTATCAGCTGCGTGATAGCAAAAGCAGC<br>GATAAAAGCGTGTGCCTGTTTACCGATTTTGATAGCCAGACCAACGTGAGCCAGAGCAAAGATAG<br>CGATGTGTATATTACCGATAAATGCGTGCTGGATATGCGCAGCATGGATTTTAAAAGCAACAGCG<br>CGGTGGCGTGGAGCAACAAAAGCGATTTTGCGTGCGCGAACGCGTTTAACAACAGCATCATTCCG<br>GAAGATACCTTTTTCCCGAGCCCGGAAAGCAGC**TAATGA**CTCGAG
RLQ3<br>beta	CAT**ATG**GGTGTTGCGCAGAGCCCGCGCTATAAAATCATTGAAAAACGTCAGAGCGTGGCCTTTTG<br>GTGCAACCCGATTAGCGGCCACGCGACCCTGTATTGGTATCAACAGATTCTGGGCCAGGGTCCGA<br>AACTGCTGATTCAGTTTCAGAACAATGGCGTGGTTGATGACAGCCAGCTGCCGAAAGATCGCTTT<br>AGCGCGGAACGTCTGAAAGGCGTGGATAGCACCCTGAAAATCCAGCCGGCCAAACTGGAAGATAG<br>CGCGGTGTATCTGTGTGCGAGCAGCCTGGGCGGTGCGGGTGGCGCCGATACCCAGTACTTTGGTC<br>CGGGTACCCGTCTGACCGTGCTGGAAGATCTGAAAAACGTGTTTCCGCCGGAAGTGGCGGTGTTT<br>GAACCGAGCGAAGCGGAAATTAGCCATACCCAGAAAGCGACCCTGGTGTGCCTGGCGACCGGTTT<br>TTATCCGGATCATGTGGAACTGAGCTGGTGGGTGAACGGCAAAGAAGTGCATAGCGGCGTGTGCA<br>CCGATCCGCAGCCGCTGAAAGAACAGCCGGCGCTGAACGATAGCCGTTATGCGCTGAGCAGCCGT<br>CTGCGTGTGAGCGCGACCTTTTGGCAGAACCCGCGCAACCATTTTCGCTGCCAAGTTCAGTTTTA<br>CGGACTGTCGGAAAACGATGAATGGACCCAGGATCGTGCGAAACCGGTGACCCAGATTGTGAGCG<br>CGGAAGCGTGGGGCCGCGCGGAT**TAATGA**CTCGAG
YLQ7<br>alpha	CAT**ATG**CAGAAAGAGGTGGAACAAAACAGCGGTCCGCTGAGCGTTCCGGAGGGTGCGATTGCGAG<br>CCTGAACTGCACCTACAGCGATCGTGGTAGCCAGAGCTTCTTTTGGTACCGTCAATATAGCGGCA<br>AAAGCCCGGAGCTGATCATGTTCATTTATAGCAACGGTGACAAGGAAGATGGCCGTTTTACCGCG<br>CAGCTGAACAAAGCGAGCCAATACGTGAGCCTGCTGATTCGTGACAGCCAGCCGAGCGATAGCGC<br>GACCTATCTGTGCGCGGTTAACCGTGACGATAAGATCATTTTCGGTAAAGGCACCCGTCTGCACA<br>TCCTGCCGAACATTCAGAACCCGGACCCGGCGGTGTACCAACTGCGTGACAGCAAGAGCAGCGAT<br>AAAAGCGTGTGCCTGTTCACCGACTTTGATAGCCAGACCAACGTTAGCCAAAGCAAGGACAGCGA<br>TGTGTATATCACCGACAAATGCGTTCTGGATATGCGTAGCATGGACTTTAAGAGCAACAGCGCGG<br>TTGCGTGGAGCAACAAAAGCGATTTCGCGTGCGCGAACGCGTTTAACAACAGCATCATTCCGGAG<br>GACACCTTCTTTCCGAGCCCGGAAAGCAGC**TAATGA**CTCGAG
YLQ7<br>beta	CAT**ATG**GACACCGGTGTGAGCCAGAACCCGCGTCACAAGATCACCAAACGTGGCCAAAACGTTAC<br>CTTCCGTTGCGATCCGATTAGCGAGCACAACCGTCTGTACTGGTATCGTCAGACCCTGGGTCAAG<br>GCCCGGAATTCCTGACCTACTTTCAGAACGAGGCGCAACTGGAAAAGAGCCGTCTGCTGAGCGAC<br>CGTTTCAGCGCGGAGCGTCCGAAAGGTAGCTTTAGCACCCTGGAGATCCAGCGTACCGAACAAGG<br>CGACAGCGCGATGTACCTGTGCGCGAGCAGCCCGGATATTGAGCAGTATTTTGGTCCGGGTACCC<br>GTCTGACCGTGACCGAAGACCTGAAGAACGTTTTCCCGCCGGAAGTGGCGGTTTTTGAACCGAGC<br>GAGGCGGAAATCAGCCACACCCAAAAAGCGACCCTGGTGTGCCTGGCGACCGGTTTTTATCCGGA<br>TCACGTGGAGCTGTCCTGGTGGGTTAACGGCAAGGAAGTGCATAGCGGCGTTTGCACCGACCCGC<br>AGCCGCTGAAAGAGCAACCGGCGCTGAACGATAGCCGTTATGCGCTGAGCAGCCGTCTGCGTGTG<br>AGCGCGACCTTTTGGCAGAACCCGCGTAACCACTTCCGTTGCCAGGTTCAATTTTATGGTCTGAG<br>CGAGAACGACGAATGGACCCAGGATCGTGCGAAGCCGGTGACCCAAATTGTTAGCGCGGAAGCGT<br>GGGGCCGTGCGGAT**TAATGA**CTCGAG

Start and stop codons are shown in bold. Sequences coding for upstream (Nde I) and downstream (Xho I) cloning sites are underlined.

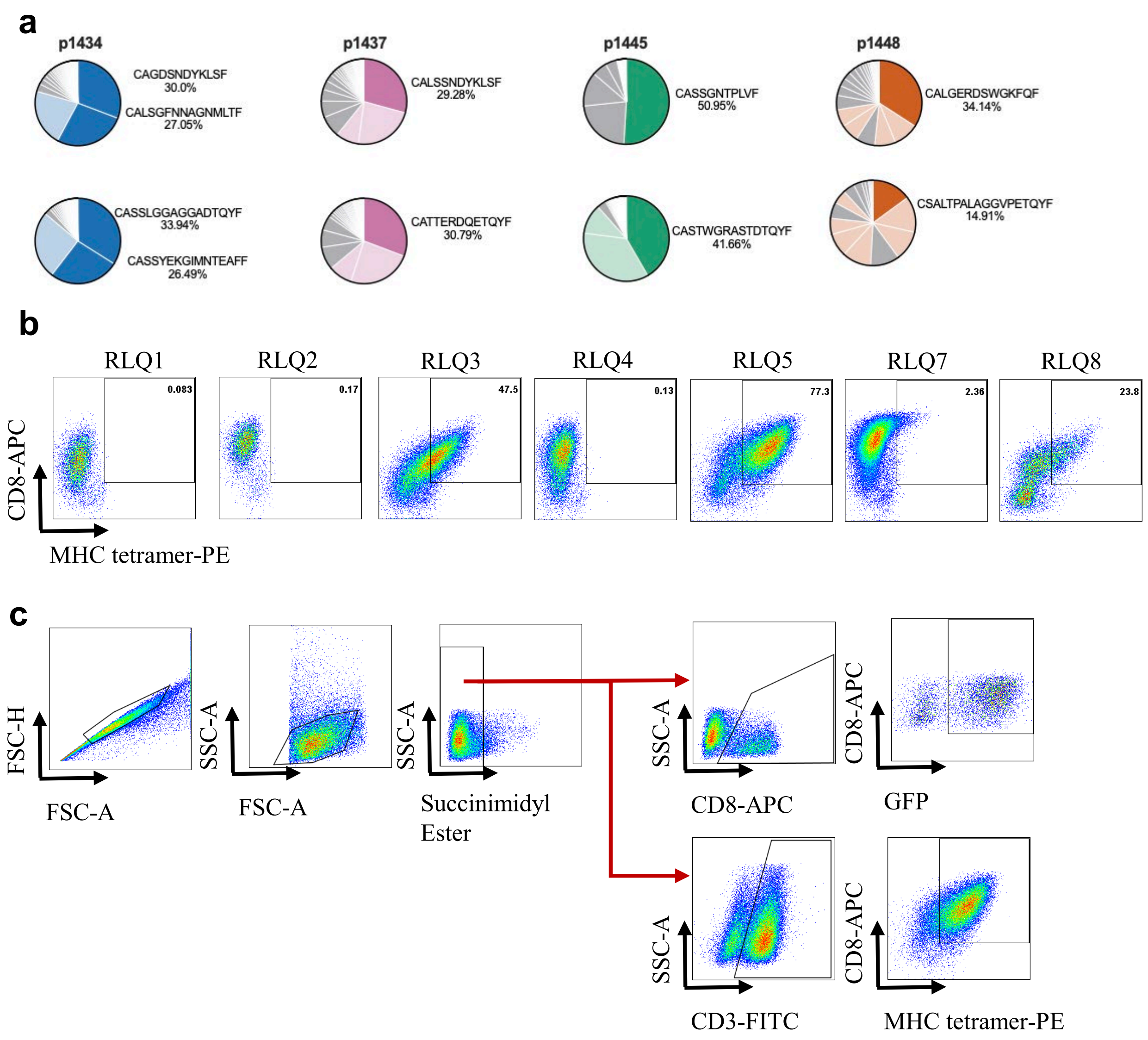

Supplementary Figure 1. Pairing of α and β chains of RLQ-specific TCRs. **(a)** The share of α (top) and β (bottom) chains in MHC tetramer$^+$ fraction in different samples. Patient ID is indicated above. Color indicates the chains which were strongly (R10-fold) and significantly ($p < 10^8$, Fisher's exact test) enriched in MHC tetramer$^+$ fraction in the previous work (26). Darker color indicates the chains that were cloned and analyzed in this study. **(b)** MHC tetramer staining of the putative RLQ-specific clones CD3$^+$ cells are gated. Numbers indicate the percentage of cells in the MHC tetramer$^+$ gate. **(c)** Reproduction of the gating strategy defining activated CD8$^+$GFP$^+$ cells or RLQ-specific CD3$^+$ cells.

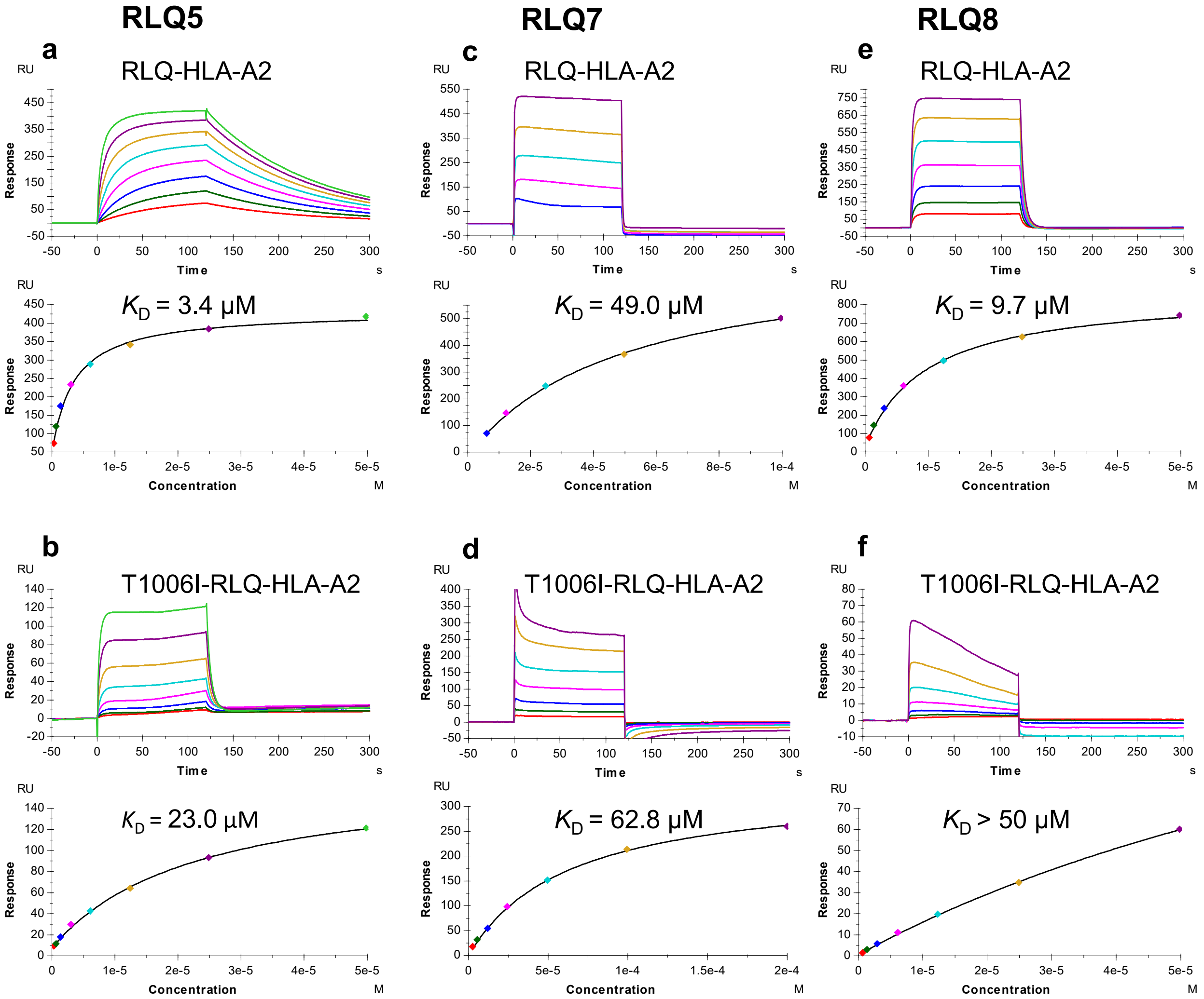

Supplementary Figure 2. SPR analysis of RLQ-specific TCRs binding to RLQ epitopes and T1006I variant. **(a)** (upper) TCR RLQ5 at concentrations of 0.39, 0.78, 1.56, 3.12, 6.25, 12.5, 25.0, and 50.0 μM was injected over immobilized RLQ–HLA-A2 (1200 RU). (lower) Fitting curve for equilibrium binding that resulted in a K_D of 3.4 μM. **(b)** (upper) TCR RLQ5 at concentrations of 0.39, 0.78, 1.56, 3.12, 6.25, 12.5, 25.0, and 50.0 μM was injected over immobilized T1006I–HLA-A2 (1200 RU). (lower) Fitting curve for equilibrium binding that resulted in a K_D of 23.0 μM. **(c)** (upper) TCR RLQ7 at concentrations of 6.25, 12.5, 25.0, 50.0, and 100 μM was injected over immobilized RLQ–HLA-A2 (3000 RU). (lower) Fitting curve for equilibrium binding that resulted in a K_D of 49.0 μM. **(d)** (upper) TCR RLQ7 at concentrations of 3.12, 6.25, 12.5 25.0 50.0, 100, and 200 μM was injected over immobilized T1006I–HLA-A2 (3000 RU). (lower) Fitting curve for equilibrium binding that resulted in a K_D of 62.8 μM. **(e)** (upper) TCR RLQ8 at concentrations of 0.78, 1.56, 3.12, 6.25, 12.5, 25.0, and 50.0 μM was injected over immobilized RLQ–HLA-A2 (1200 RU). (lower) Fitting curve for equilibrium binding that resulted in a K_D of 9.7 μM. **(f)** (upper) TCR RLQ8 at concentrations of 0.78, 1.56, 3.12, 6.25, 12.5, 25.0, and 50.0 μM was injected over immobilized T1006I–HLA-A2 (1200 RU). (lower) Fitting curve for equilibrium binding. Since saturation was not reached, K_D was estimated at >50 μM.

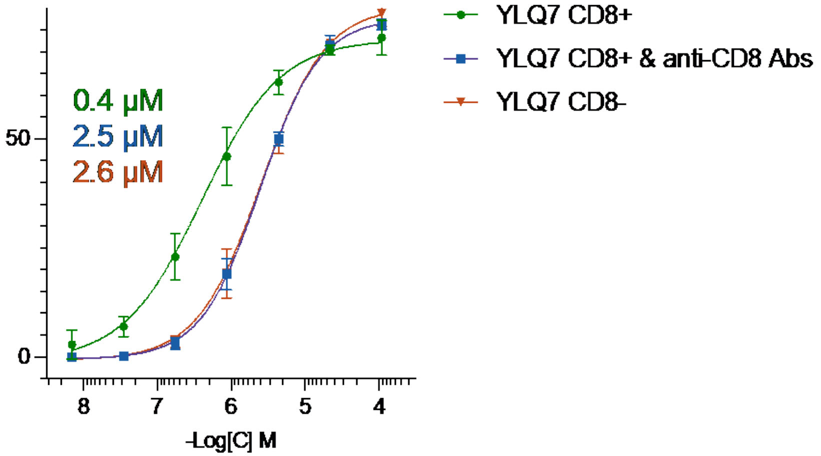

Supplementary Figure 3. Contribution of CD8 to YLQ7–pMHC binding. The J76 TPR cell lines with transgenic TCR YLQ7 with CD8 co-receptor (YLQ7 CD8+), without CD8 co-receptor (YLQ7 CD8-) or with CD8 co-receptor but in the presence of anti-CD8 blocking antibodies (YLQ7 CD8+ & anti-CD8 Abs) were co-cultivated with the K562-A*02 cell line loaded with various concentrations of the cognate peptide (n=3 independent replicates). T cell activation was measured by eGFP expression regulated by the NFAT promoter. Plotted are the mean share of $eGFP^+$ cells and SD. EC_{50} values are shown for YLQ7 with CD8 (green), without CD8 (orange), and in the presence of anti-CD8 antibodies (blue).

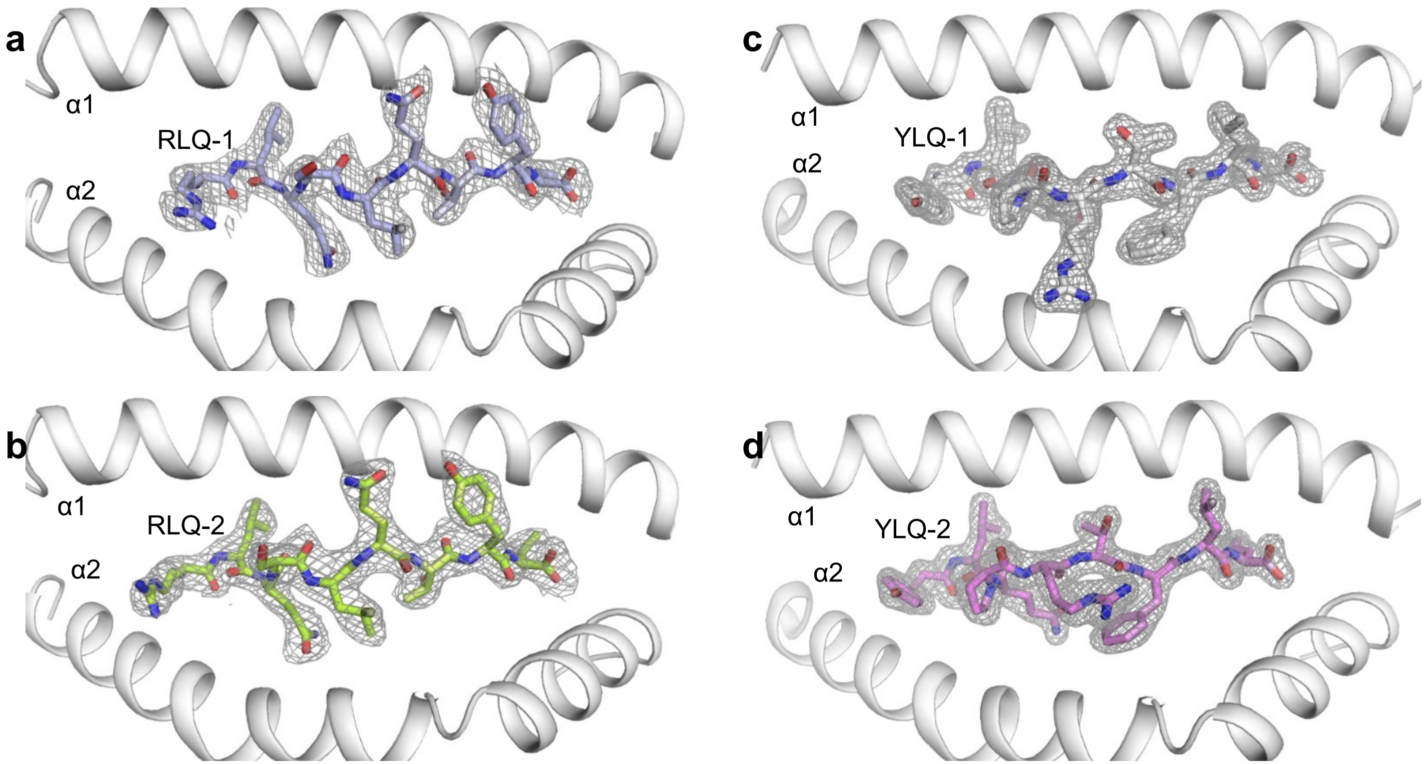

Supplementary Figure 4. (a–b) Electron density for the bound RLQ peptide in the two RLQ–HLA complexes in the asymmetric unit of the crystal. The $F_o - F_c$ omit map at 2.81 Å resolution is contoured at 1σ. The two RLQ peptides have identical conformations. **(c–d)** Electron density for the bound YLQ peptide in the two YLQ–HLA-A2 complexes in the asymmetric unit of the crystal. The $F_o - F_c$ omit map at 2.07 Å resolution is contoured at 1σ. The YLQ peptide displays two different conformations.

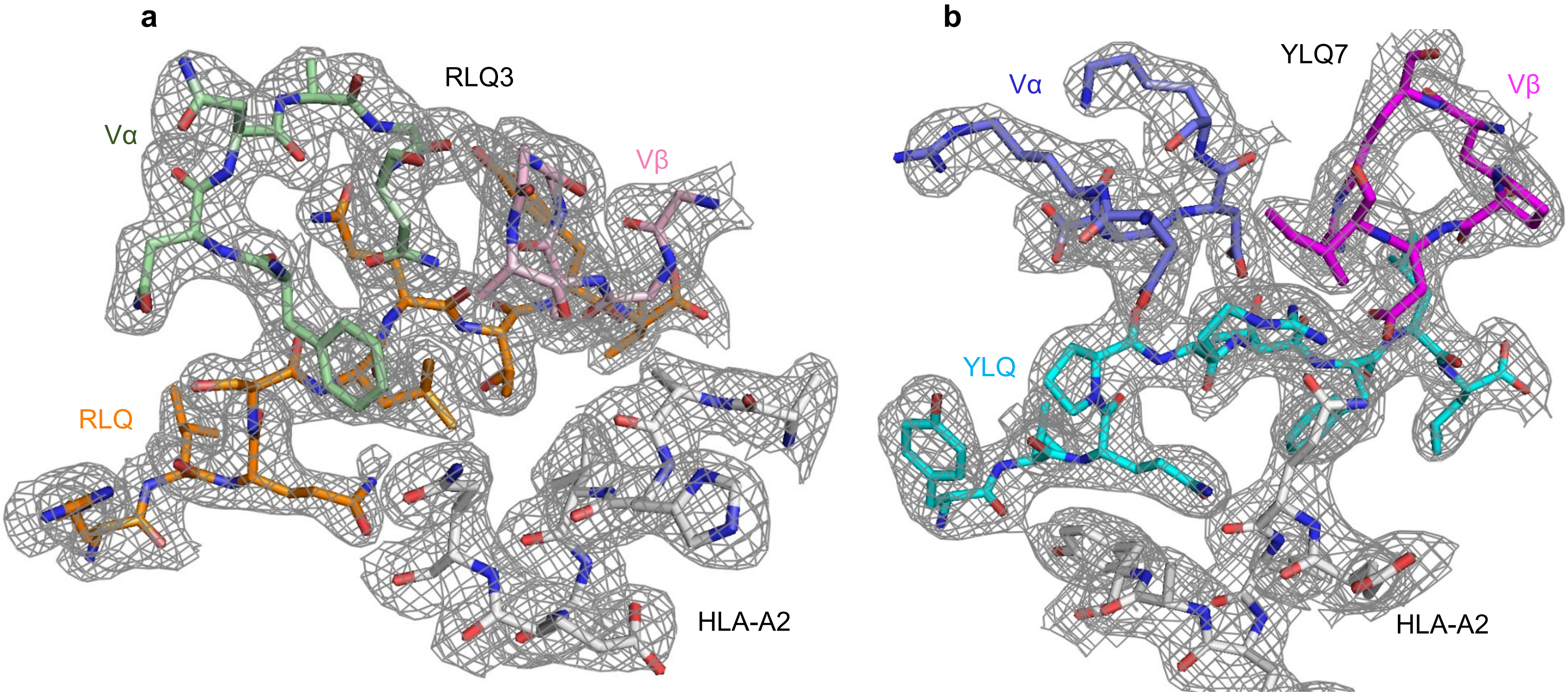

Supplementary Figure 5. (a) Electron density at the interface in the RLQ3–RLQ–HLA-A2 complex. Density from the final $2F_o - F_c$ map at 2.30 Å resolution is contoured at 1σ. **(b)** Electron density at the interface in the YLQ7–YLQ–HLA-A2 complex. Density from the final $2F_o - F_c$ map at 2.39 Å resolution is contoured at 1σ.

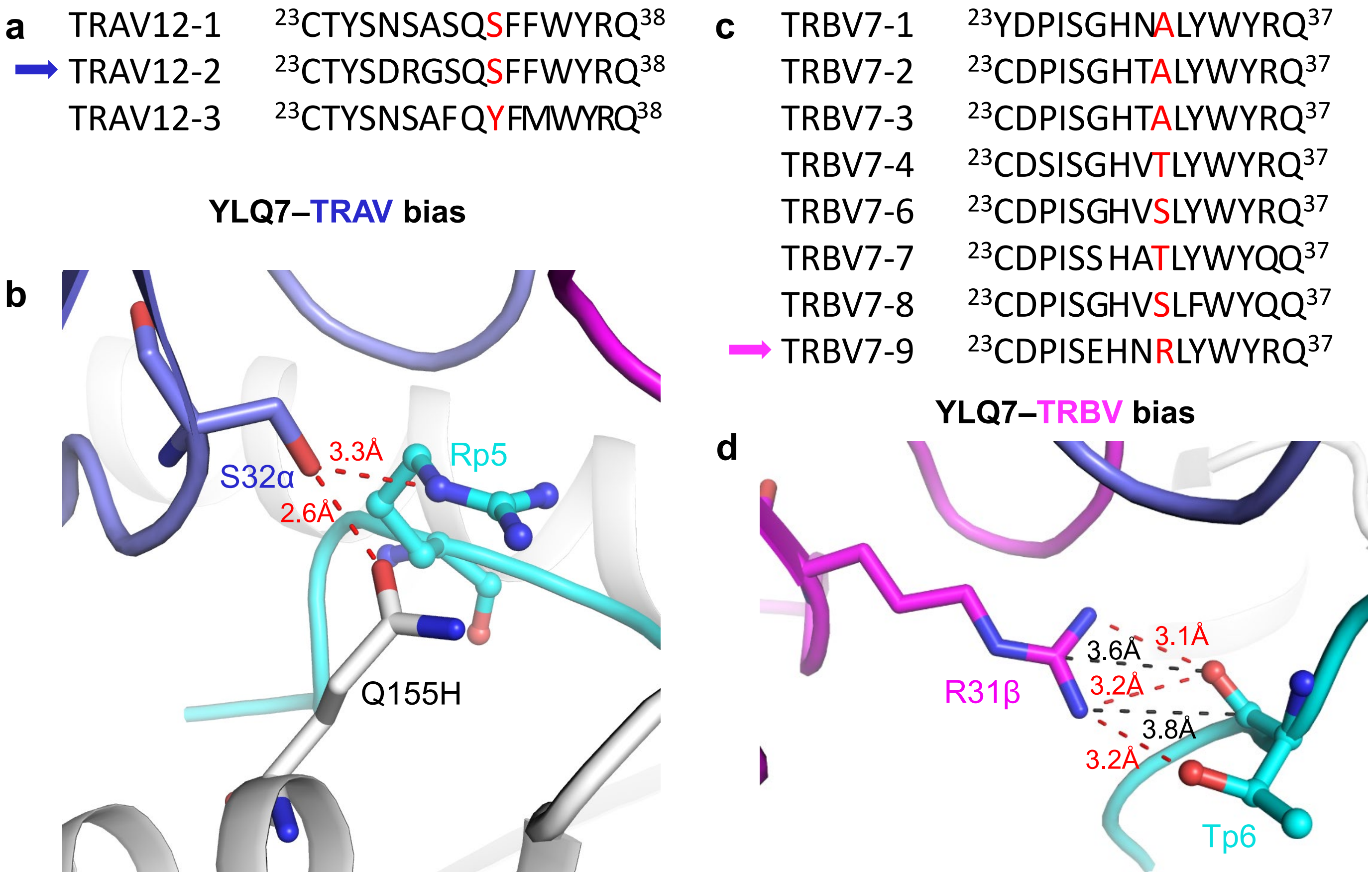

Supplementary Figure 6. Structural basis for TRAV and TRBV bias of TCR YLQ7. **(a)** Sequence alignment of CDR1α of TRAV12 family members. Blue arrow indicates CDR1α sequence of TCR YLQ7. **(b)** Ser32α of YLQ7 forms two hydrogen bonds with Q155H of HLA-A2 and P5 Arg of the YLQ peptide. These interactions would be disrupted by the bulkier Tyr32α. Hydrogen bonds are drawn as red dashed lines. **(c)** Sequence alignment of CDR1β of TRBV7 family members. Magenta arrow indicates CDR1β sequence of TCR YLQ7. **(d)** Arg31β of YLQ7 forms three hydrogen bonds and two van der Waals contacts with P6 Thr of the YLQ peptide. Other TRBV7 family members encode Ser31α, Thr31α, or Ala31α, whose smaller and uncharged side chains cannot replicate these key interactions. Hydrogen bonds are red dashed lines and van der Waals contacts are black dashed lines.

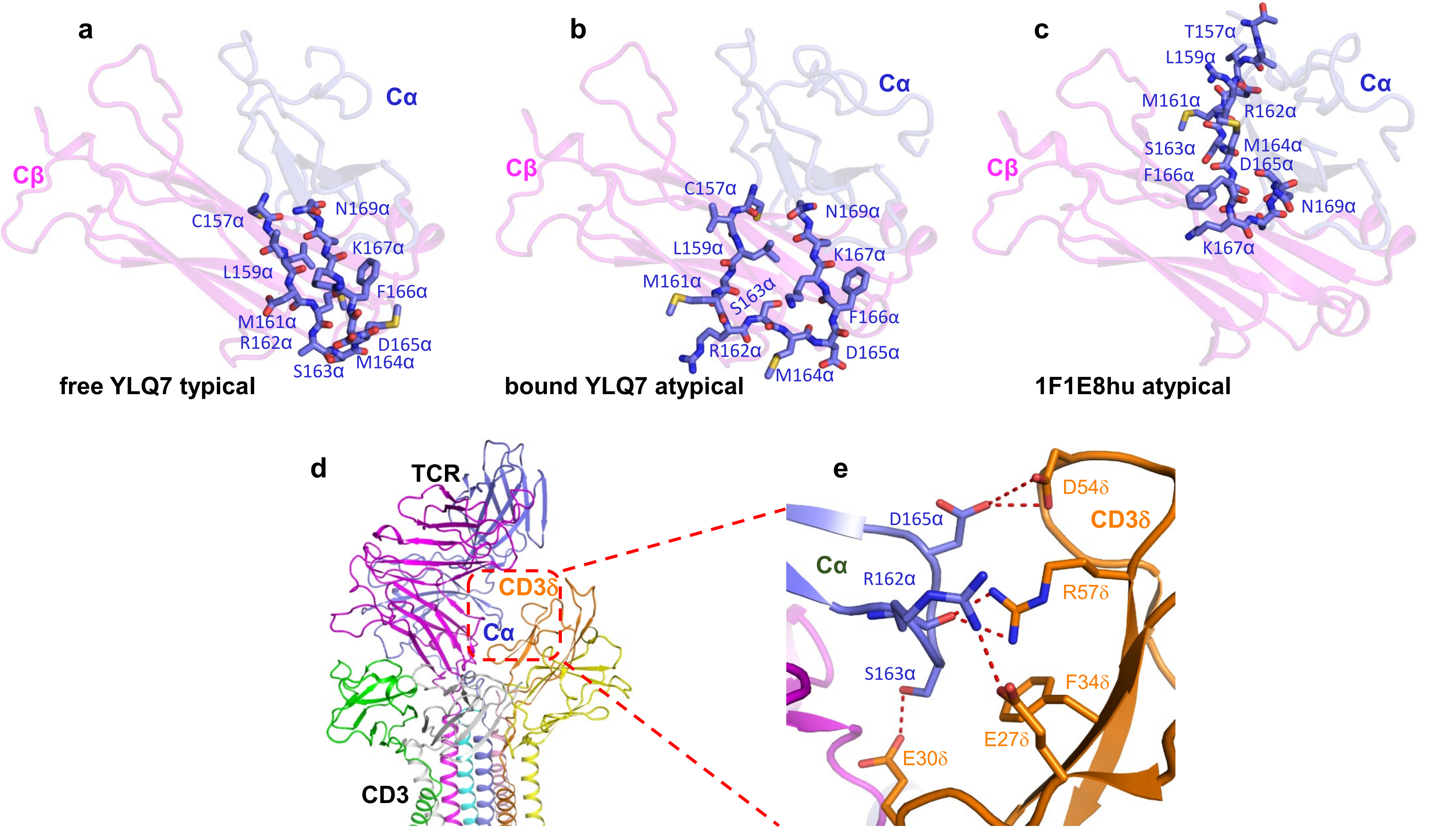

Supplementary Figure 7. Comparison of Cα domain conformations in free versus ligand-bound TCR YLQ7. **(a)** The typical Cα conformation in free TCR YLQ7 (7N1D, this work). **(b)** The atypical Cα conformation in YLQ7 bound to YLQ–HLA-A2 (7N1F, this work). **(c)** The atypical Cα conformation in TCR 1F1E8hu (3MFF) (4). (**d**) CryoEM structure of the TCR–CD3 complex (6JXR) (5). The region of contact between Cα and the CD3δ subunit of the CD3εδ heterodimer is boxed. (**e**) Close-up of interactions between Cα and CD3δ. The side chains of contacting residues are drawn in stick representation.

Supplementary References

1. Collaborative Computational Project No. 4. The CCP4 suite: programs for protein crystallography. *Acta Crystallogr. D Biol. Crystallogr.* **50**, 240-255 (1994).
2. Reynisson, B., Alvarez, B., Paul, S., Peters, B. & Nielsen, M. NetMHCpan-4.1 and NetMHCIIpan-4.0: improved predictions of MHC antigen presentation by concurrent motif deconvolution and integration of MS MHC eluted ligand data. *Nucleic Acids Res.* **48**, W449-W454 (2020).
3. Kortemme, T., Kim, D.E. & Baker, D. Computational alanine scanning of protein-protein interfaces. *Sci STKE* **2004**, pl2 (2004).
4. van Boxel, G.I., Holmes, S., Fugger, L. & Jones, E.Y. An alternative conformation of the T-cell receptor α constant region. *J. Mol. Biol.* **400**, 828-837 (2010).
5. Dong, D. et al. Structural basis of assembly of the human T cell receptor-CD3 complex. *Nature* **573**, 546-552 (2019).
