# Supplementary material for: Structural assessment of HLA-A2-restricted SARS-CoV-2 spike epitopes recognized by public and private T-cell receptors
Source: Nat Commun. 2022 Jan 10;13:19. doi: 10.1038/s41467-021-27669-8 (PMC8748687; doi:10.1038/s41467-021-27669-8)
Supplement: Supplementary file 3 — Reporting summary [file 41467_2021_27669_MOESM3_ESM.pdf]

nature portfolio

Corresponding author(s): Roy A. Mariuzza

Last updated by author(s): Sep 30, 2021

Reporting Summary

Nature Portfolio wishes to improve the reproducibility of the work that we publish. This form provides structure for consistency and transparency in reporting. For further information on Nature Portfolio policies, see our Editorial Policies and the Editorial Policy Checklist.

Statistics

For all statistical analyses, confirm that the following items are present in the figure legend, table legend, main text, or Methods section.

n/a	Confirmed	
☒	☐	The exact sample size (*n*) for each experimental group/condition, given as a discrete number and unit of measurement
☒	☐	A statement on whether measurements were taken from distinct samples or whether the same sample was measured repeatedly
☒	☐	The statistical test(s) used AND whether they are one- or two-sided<br>*Only common tests should be described solely by name; describe more complex techniques in the Methods section.*
☒	☐	A description of all covariates tested
☒	☐	A description of any assumptions or corrections, such as tests of normality and adjustment for multiple comparisons
☒	☐	A full description of the statistical parameters including central tendency (e.g. means) or other basic estimates (e.g. regression coefficient) AND variation (e.g. standard deviation) or associated estimates of uncertainty (e.g. confidence intervals)
☒	☐	For null hypothesis testing, the test statistic (e.g. *F*, *t*, *r*) with confidence intervals, effect sizes, degrees of freedom and *P* value noted<br>*Give P values as exact values whenever suitable.*
☒	☐	For Bayesian analysis, information on the choice of priors and Markov chain Monte Carlo settings
☒	☐	For hierarchical and complex designs, identification of the appropriate level for tests and full reporting of outcomes
☒	☐	Estimates of effect sizes (e.g. Cohen's *d*, Pearson's *r*), indicating how they were calculated

Our web collection on statistics for biologists contains articles on many of the points above.

Software and code

Policy information about availability of computer code

Data collection	HKL3000: Minor, W., Cymborowski, M., Otwinowski, Z. & Chruszcz, M. HKL-3000: the integration of data reduction and structure solution--from diffraction images to an initial model in minutes. Acta Crystallogr. D Biol Crystallogr. 62, 859-866 (2006)
Data analysis	CCP4: Collaborative Computational Project No. 4. The CCP4 suite: programs for protein crystallography. Acta Crystallogr. D Biol. Crystallogr. 50, 240-255 (1994)<br>Phaser: Storoni, L.C., McCoy, A.J. & Read, R.J. Likelihood-enhanced fast rotation functions. Acta Crystallogr D Biol Crystallogr. 60, 432-438 (2004)<br>Phenix: Afonine, P.V. et al. Towards automated crystallographic structure refinement with phenix.refine. Acta Crystallogr D Biol Crystallogr. 68, 352-367 (2012)<br>Refmac: Murshudov, G.N., Vagin, A.A. & Dodson, E.J. Refinement of macromolecular structures by the maximum-likelihood method. Acta Crystallogr D Biol Crystallogr. 53, 240-255 (1997)<br>Coot: Emsley, P., Lohkamp, B., Scott, W.G. & Cowtan, K. Features and development of Coot. Acta Crystallogr D Biol Crystallogr. 66, 486-501 (2010)<br>PyMOL (https://pymol.org/)<br>BIAevaluation 3.1 (GE Healthcare)<br>MACSQuant Analyzer 10 (Miltenyi Biotec)<br>by FlowJo (version 10.6.2)<br>Prizm Software(https://www.graphpad.com/)

For manuscripts utilizing custom algorithms or software that are central to the research but not yet described in published literature, software must be made available to editors and reviewers. We strongly encourage code deposition in a community repository (e.g. GitHub). See the Nature Portfolio guidelines for submitting code & software for further information.

Data

Policy information about availability of data

All manuscripts must include a data availability statement. This statement should provide the following information, where applicable:

- Accession codes, unique identifiers, or web links for publicly available datasets
- A description of any restrictions on data availability
- For clinical datasets or third party data, please ensure that the statement adheres to our policy

Atomic coordinates and structure factors have been deposited in the Protein Data Bank under accession codes 7N1A (YLQ–HLA-A2), 7N1B (RLQ–HLA-A2), 7N1C (RLQ3), 7N1D (YLQ7), 7N1E (RLQ3–RLQ–HLA-A2), and 7N1F (YLQ7–YLQ–HLA-A2).

Field-specific reporting

Please select the one below that is the best fit for your research. If you are not sure, read the appropriate sections before making your selection.

- [x] Life sciences
- [ ] Behavioural & social sciences
- [ ] Ecological, evolutionary & environmental sciences

For a reference copy of the document with all sections, see nature.com/documents/nr-reporting-summary-flat.pdf

Life sciences study design

All studies must disclose on these points even when the disclosure is negative.

Sample size	n/a
Data exclusions	n/a
Replication	n/a
Randomization	n/a
Blinding	n/a

Reporting for specific materials, systems and methods

We require information from authors about some types of materials, experimental systems and methods used in many studies. Here, indicate whether each material, system or method listed is relevant to your study. If you are not sure if a list item applies to your research, read the appropriate section before selecting a response.

Materials & experimental systems

n/a	Involved in the study	
[ ]	[x]	Antibodies
[ ]	[x]	Eukaryotic cell lines
[x]	[ ]	Palaeontology and archaeology
[x]	[ ]	Animals and other organisms
[x]	[ ]	Human research participants
[x]	[ ]	Clinical data
[x]	[ ]	Dual use research of concern

Methods

n/a	Involved in the study	
[x]	[ ]	ChIP-seq
[ ]	[x]	Flow cytometry
[x]	[ ]	MRI-based neuroimaging

Antibodies

Antibodies used	CD8-APC (SK1), BD, Cat 345775, lot 9329558<br>CD3-APC, BD, cat. 345767 lot. 324347 (for tetramer staining)<br>CD3 -FITC, BD,cat. 345764 lot.9029609
Validation	Validated by the manufacturer

Eukaryotic cell lines

Policy information about cell lines

Cell line source(s)	J76 TPR TCR KO cell line 43. Rosskopf, S. et al. A Jurkat 76 based triple parameter reporter system to evaluate TCR functions

	and adoptive T cell strategies. Oncotarget 9, 17608-17619 (2018); K562 cell line (ATCC)
Authentication	STR profiling
Mycoplasma contamination	all cell lines tested negative for mycoplasma
Commonly misidentified lines (See ICLAC register)	n/a

Flow Cytometry

Plots

Confirm that:

- [x] The axis labels state the marker and fluorochrome used (e.g. CD4-FITC).
- [x] The axis scales are clearly visible. Include numbers along axes only for bottom left plot of group (a 'group' is an analysis of identical markers).
- [x] All plots are contour plots with outliers or pseudocolor plots.
- [x] A numerical value for number of cells or percentage (with statistics) is provided.

Methodology

Sample preparation	Surface staining
Instrument	MACSQuant Analyzer 10
Software	FlowJo
Cell population abundance	J76 cell cultures were 60-99% CD8 or CD3 positive
Gating strategy	J76 cells were gated by FSC-H/SSC-A for singlets, FSC-A/SSC-A for lymphocytes, SSC-A/LD for live cells, CD8+ or CD3+ (for CD8-line), eGFP+ gate was set by the negative control

- [x] Tick this box to confirm that a figure exemplifying the gating strategy is provided in the Supplementary Information.
